# Non-stimulated adrenal venous sampling using Dyna computed tomography in patients with primary aldosteronism

**DOI:** 10.1038/srep37143

**Published:** 2016-11-23

**Authors:** Chin-Chen Chang, Bo-Ching Lee, Kao-Lang Liu, Yeun-Chung Chang, Vin-Cent Wu, Kuo-How Huang

**Affiliations:** 1 Department of Medical Imaging, National Taiwan University Hospital and National Taiwan University College of Medicine, Taipei, Taiwan; 2 Department of Internal Medicine, National Taiwan University Hospital and National Taiwan University College of Medicine, Taipei, Taiwan; 3 Department of Urology, National Taiwan University Hospital and National Taiwan University College of Medicine, Taipei, Taiwan

## Abstract

In this retrospective study, we aimed to examine the effect of applying Dyna computed tomography (CT) on the success rate of adrenal venous sampling (AVS) without adrenocorticotropic hormone stimulation. A total of 100 consecutive patients with primary aldosteronism who underwent AVS between May 2012 and July 2015 were enrolled. In all the cases, Dyna CT was used in AVS to validate catheter position in the right adrenal vein. A selectivity index (cortisol_adrenal vein_ /cortisol_inferior vena cava_) of ≥2.0 of both adrenal veins were required for successful AVS. Dyna CT indicated misplaced catheters in 16 patients; of these patients, 75% (12/16) eventually had successful right AVS after catheter repositioning. The success rate of initial sampling at the right adrenal vein was 76% (76/100), which increased to 88% (88/100) after Dyna CT was applied (*p* < 0.001). The most common inadvertently catheterised vessels detected using Dyna CT were the accessory hepatic veins (56.3%, 9/16), followed by the renal capsular veins (37.5%, 6/16). The overall success rate of non-stimulated AVS using Dyna CT was 87% (87/100). Thus, the application of Dyna CT further increased the success rate of non-stimulated AVS.

Primary aldosteronism (PA) is currently considered as the most common cause of secondary hypertension, and accounts for 5–11% of unselected hypertensive patients^1^,^2^,^3^. The causes underlying PA include an aldosterone-producing adenoma, unilateral adrenal hyperplasia, and bilateral idiopathic adrenal hyperplasia^2^,^3^,^4^. Moreover, the treatment options primarily depend on whether the PA is unilateral or bilateral. In patients with unilateral hyperaldosteronism, surgical treatment is commonly indicated, as 30–60% of patients can be cured of hypertension after unilateral adrenalectomy^5^,^6^. In contrast, patients with bilateral PA usually receive long-term medical treatment, such as mineralocorticoid receptor antagonist therapy^7^.

Cross-sectional adrenal imaging has been shown to be unreliable for the lateralisation of PA^8^,^9^,^10^. Therefore, adrenal venous sampling (AVS) is currently recognised as the gold standard for determining the laterality of PA through the direct sampling of aldosterone secretion. Accordingly, the Endocrine Society guidelines recommend the use of AVS for distinguishing between unilateral and bilateral hyperaldosteronism^10^. However, the catheterisation of the right adrenal vein is difficult due to its small size, which makes AVS a technically challenging procedure with reported success rates ranging from 55% to 97% 8,11,12,13.

Dyna computed tomography (CT) has recently been advocated as a powerful tool for assisting catheterisation of the right adrenal vein^11^,^14^. As the sampling of the right adrenal vein is the most technically challenging part of AVS, Dyna CT is primarily used to confirm right adrenal venous catheterisation in these cases^11^. Nevertheless, only a few studies have focused on the value of Dyna CT in cases of non-stimulated AVS. In the present study, we aimed to investigate the effect of applying Dyna CT on the success rate of adrenal venous sampling (AVS) without adrenocorticotropic hormone (ACTH) stimulation.

## Methods

This retrospective study was compliant with the Health Insurance Portability and Accountability Act, and was approved by the institutional review board of the National Taiwan University Hospital. Between May 2012 and July 2015, 100 consecutive patients with PA underwent Dyna CT-assisted AVS at our institution. Dyna CT images and laboratory data were recorded in the Taiwan Primary Aldosteronism Investigation (TAIPAI) database.

### Identification of PA

No antihypertensive medications were administered for at least 21 days prior to the confirmatory test. Patients with markedly high blood pressure were given diltiazem and/or doxazosin, if required^15^. PA was diagnosed in cases that fulfilled the following 3 criteria: aldosterone to renin ratio (ARR) >35; TAIPAI score >60% 16; and post-saline loading plasma aldosterone concentration of >10 ng/dl^17^.

### AVS protocol

A radiologist (C.C.C.) with 6 years of experience with AVS performed all the procedures. Informed consent was obtained from all patients prior to AVS. To avoid any bias introduced as a result of the aldosterone circadian rhythm^18^, all AVS procedures were conducted in the morning. Patients were encouraged to maintain a supine position before the AVS procedure for at least 1 h. No ACTH stimulation was performed before or during AVS. We used real-time ultrasound guidance for puncture of the right femoral vein, and a 5-French vascular sheath was placed using the Seldinger technique. In most cases, a 4-French C1 catheter with 2 side holes (Torcon NB, Cook Medical, Bloomington, U.S.A.) was used for venous sampling of the both adrenal veins and the inferior vena cava (IVC). Before sampling both the adrenal veins, the catheter tip position was carefully confirmed via venography, through the gentle injection of a small amount of diluted contrast medium (Omnipaque 350, GE healthcare, Carrigtohill, Ireland). If the catheter was in an unstable position (n = 9), a microcatheter (Renegade 2.5Fr, Boston Scientific, Boston, U.S.A.) was navigated into the right adrenal vein for better stabilisation. Dyna CT was used to verify right adrenal venous catheterisation in all cases. To minimise the risk of catheter displacement during sampling, patients were asked to avoid deep breathing, forceful coughing, and talking for a short period. The catheter tip position was reconfirmed via venography after the sampling was completed.

### Dyna CT protocol

A ceiling-mounted angiography system (Zeego Artis, Siemens, Erlangen, Germany) was used for all Dyna CT scans. Each patient was instructed to place their arms over their head to reduce streak artifacts on Dyna CT images. The isocentre was set at an anteroposterior and lateral position to ensure the presence of the right adrenal vein in the field of view (FOV). Thereafter, a test run was performed to ensure that no collision occurred. The contrast medium was diluted 3 times to avoid the production of streak artifacts following its injection. The actual Dyna CT image was acquired 2 s after the start of contrast medium injection (at a rate of 0.5–1.0 mL/s for 8 s). The rotation time for Dyna CT was 6 s, whereas the detector moved at 45°/s. The patients were told to hold their breath and to avoid any intentional inspiration or expiration as soon as the contrast medium was injected. The source power was 90 kVp, and the FOV was 48 cm, with a voxel matrix of 512 × 512.

### Dyna CT interpretation

**Figure 1:**
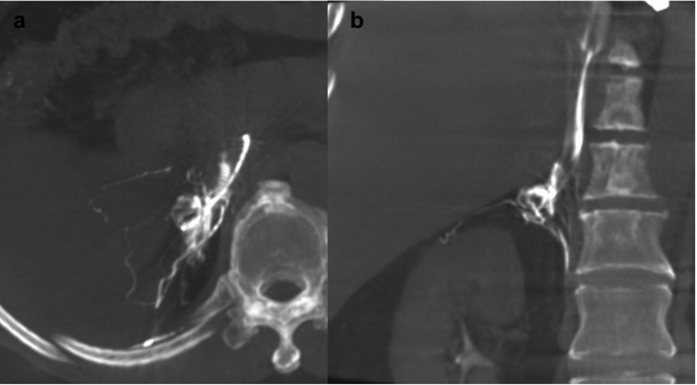
A 60-year-old man with primary aldosteronism (PA) and a right adrenal nodule. The right adrenal gland was opacified on a maximum intensity projection image on Dyna computed tomography in the transverse plane (**a**) and coronal plane (**b**). Adrenal venous sampling was successful and the lateralisation examination indicated bilateral PA.

Dyna CT images were processed and interpreted on a Siemens workstation on site (Advantage workstation, Siemens, Erlangen, Germany). Three-dimensional multiplanar reconstructed images were used to verify right adrenal venous catheterisation, with free-adjustment of the window level, window centre, and magnification, as needed (Fig. 1). If the right adrenal vein was not opacified, the misplaced catheters were repositioned. To avoid excessive radiation exposure, Dyna CT was generally not repeated after catheter repositioning, and the position of the catheter was confirmed via venography. However, in cases with undetermined Dyna CT findings such as opacification of renal or adrenal capsular vein, repeated Dyna CT was performed at the radiologist’s discretion.

### Selectivity and lateralisation indices of AVS

A successful AVS is considered when the sampled adrenal cortisol concentration is similar or twofold greater than the sampled IVC cortisol concentration—also termed as the selectivity index^19^. Thus, a selectivity index cut-off value of ≥2.0 was used in the present study. After confirming bilateral AVS success, the lateralisation of the PA was determined based on a lateralisation index of ≥2.0; this index is estimated as the ratio of the aldosterone/cortisol (A/C) concentration on the dominant side with the A/C concentration on the contralateral side.

### Statistical analysis

All statistical analyses were performed using MedCalc statistical software (MedCalc version 15.4.0.0, Frank Schoonjans, Mariakerke, Belgium). The difference between categorical variables was compared using the Χ^2^ test, whereas the difference between independent continuous variables was compared using Mann–Whitney’s U test. The difference in the success rate before and after Dyna CT application was assessed using McNemar’s test. For all statistical analyses, a *p* value of <0.05 was considered statistically significant.

## Results

A total of 100 patients (51 men and 49 women; mean age, 52 years; age range, 31–73 years) were included in the analysis. Detailed patient characteristics are summarised in Table 1.

### Result of AVS

The overall success rate of Dyna CT-assisted AVS was 87% (87/100), including an individual success rate of 88% for the right side and 97% for the left side. No significant difference was observed in the patient characteristics and procedural time between the successful and failed AVS groups. The overall procedural time was 49.5 ± 21.3 min. With regard to lateralisation in AVS, unilateral hyperaldosteronism was identified in 44 patients (44%, 44/100), including 35 (35%, 35/100) with lateralisation towards the right and 9 (9%, 9/100) with lateralisation towards the left. However, lateralisation could not be observed in 1 patient (1%, 1/100) with successful AVS due to a laboratory error. Contrast extravasation in the right adrenal gland occurred in 1 patient (1%, 1/100) who developed mild flank soreness after contrast injection and had an uneventful recovery. The radiation dose was 673.9 ± 613.8 mGy, contributed by the fluoroscopy and Dyna CT. A detailed comparison between our study and the previous studies with similar purpose were shown in Table 2.

### Comparison of success rates before and after Dyna CT

**Figure 2:**
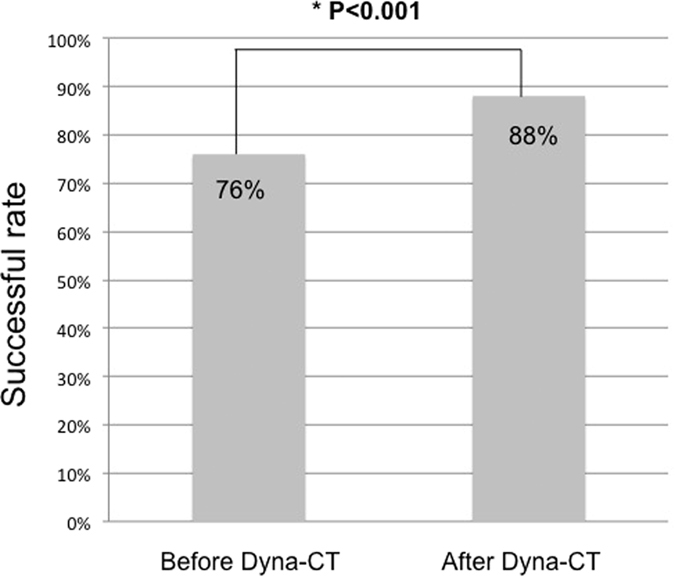
The success rate of non-stimulated adrenal venous sampling (AVS) before and after incorporating Dyna computed tomography (CT). The success rate of the initial sampling during right AVS was 76% (76/100), which increased to 88% (88/100) after Dyna CT was incorporated (*p* < 0.001).

**Figure 3:**
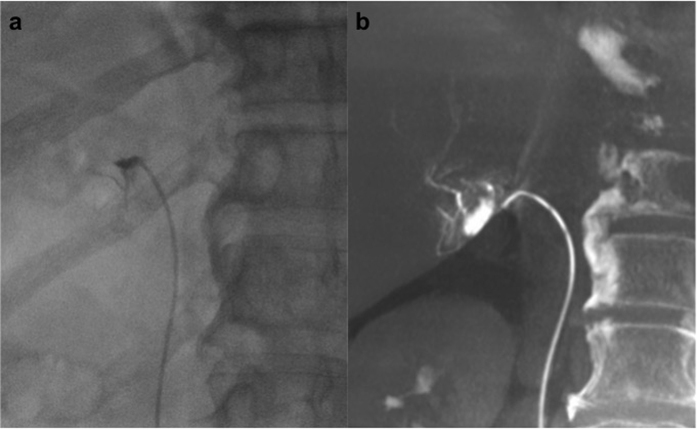
A 61-year-old man with primary aldosteronism (PA) and a right adrenal nodule. (**a**) The venogram pattern of the accessory hepatic vein was similar to that of the right adrenal vein. (**b**) Dyna computed tomography exhibited focal opacification of the liver parenchyma, with venous drainage into the accessory hepatic vein.

**Figure 4:**
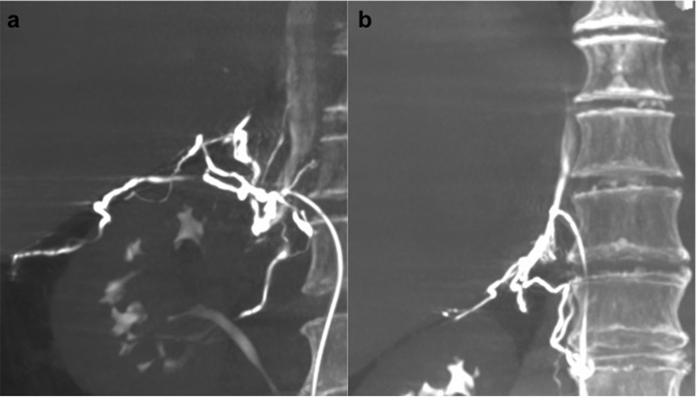
A 57-year-old man with primary aldosteronism and bilateral adrenal nodules. (**a**) On Dyna computed tomography maximum intensity projection imaging in the coronal plane, the renal capsular vein was opacified with the venous orifice at the T11/12 intervertebral level. (**b**) After adjustment of the catheter position, the right adrenal vein was opacified with the venous orifice at the mid T11 vertebral level.

**Figure 5:**
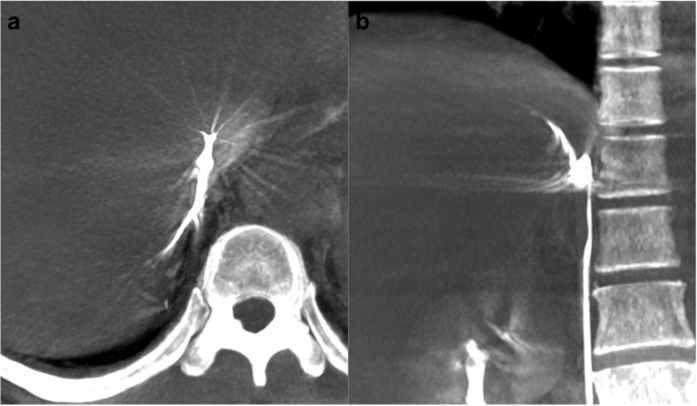
A 48-year-old woman with primary aldosteronism and a right adrenal nodule. The right inferior phrenic vein was shown on Dyna computed tomography maximum intensity projection imaging in the transverse plane (**a**) and coronal plane (**b**).

Dyna CT was conducted to confirm the catheter position in the right adrenal vein in all cases. The success rate of initial sampling in the right adrenal vein was 76% (76/100), which increased to 88% (88/100) after Dyna CT and on-site re-sampling (*p* < 0.001; Fig. 2). Catheter misplacement was detected by Dyna CT in 16% (16/100) patients; of these patients, 75% (12/16) eventually had successful sampling after catheter repositioning. On the Dyna CT images, the misplaced catheters were located in the accessory hepatic vein in 8 patients (56.3%, 9/16; Fig. 3), renal capsular vein in 5 patients (37.5%, 6/16; Fig. 4), and inferior phrenic vein in 1 patient (6.3%, 1/16; Fig. 5).

## Discussion

AVS is currently considered as the gold standard for differentiating between unilateral and bilateral hyperaldosteronism^10^. Considering the recently growing interest in the detection and diagnosis of PA^20^, AVS is expected to be more commonly used, despite its variable successful rate. However, the reliability of the technical performance of AVS is crucial for avoiding repeated AVS procedures. In the present study, we found that the success rate of non-stimulated AVS could be improved after incorporating Dyna CT.

In our study, AVS was performed without ACTH stimulation in all cases, and a cut-off value of ≥2 was used for both the selectivity and lateralisation indices, in accordance with the expert consensus statement reported by Rossi *et al*.^19^. At present, almost 50% of the medical centres worldwide use non-stimulated AVS for PA patients^21^, and the use of ACTH stimulation prior to AVS remains controversial. In fact, ACTH stimulation prior to AVS may lead to erroneous lateralisation results, and no conclusive evidence has been obtained regarding its superiority in terms of AVS outcomes^19^,^22^.

Recently, Mailhot *et al*.^23^ compared the sensitivity and specificity of different selectivity criteria for basal cortisol and aldosterone levels in 160 patients who underwent AVS with cosyntropin stimulation. The authors found that combined Aldosterone_adrenal vein_/Aldosterone_IVC_ and Cortisol_adrenal vein_/Cortisol_IVC_ values (cut-off value ≥ 2) had the best sensitivity for non-stimulated AVS, without any loss of specificity. If this selectivity index criteria by Mailhot *et al* was incorporated into the present analysis, the overall success rate would further increase to 90% (90/100), with 3 new successful cases of right AVS.

Traditionally, the AVS procedure involves the cannulation of the suspected adrenal vein under two-dimensional fluoroscopic guidance. The position of the catheter is confirmed via adrenal venography with the manual injection of a small amount of contrast. However, venography is non-specific for right adrenal vein cannulation due to the high failure rate. Nevertheless, the difference in enhancement between the enhanced vascular structure and peripheral tissue facilitates easy confirmation of catheter position by Dyna CT or other C-arm CT methods; this also improves the success rate for sampling of the right adrenal vein in AVS^11^,^24^,^25^,^26^. Onozawa *et al*.^11^ reported a success rate of 95.7% for ACTH-stimulated AVS using intraprocedural CT in a study of 140 patients. The general detection rate for misplaced catheters on intraprocedural CT was approximately 10–20%, which was similar to our results.

Catheter stabilisation is important during Dyna CT-assisted AVS. Patient movement, deep breathing, and talking increased the risk of catheter dislodgement, particularly in cases where the right adrenal vein is small or cases with a short trunk. In the present study, patients were instructed to maintain shallow breathing during the procedure, as deep breathing may lead to catheter dislodgement. Conversation with the patient was also discouraged after the catheter was placed in the right adrenal vein. In 9% patients, a microcatheter was navigated into the right adrenal vein for better stabilisation.

There were 8 patients had failed right-sided AVS despite opacified right adrenal vein on Dyna CT, and the possible cause was contamination of the sampled blood from the IVC. Although venography and Dyna could clearly indicate the right adrenal gland, the catheter tip could be occasionally wedged and blocked inside the tiny venous structure. The drawing of blood in these case required strenuous effort, causing admixture of the sampled blood with the blood of IVC through the sidehole of the catheter. Moreover, a short common venous trunk may result in the catheter sideholes being left in the IVC, which could lead to dilution of the sampling with peripheral blood. These occurrences could additionally lead to insufficient cortisol levels in the sampling. For the patients (n = 3) with unsuccessful AVS of the left side, contamination from the left inferior phrenic veins due to inadequate catheter advancement was the speculated cause for failure. Four patients in our study had unsuccessful right AVS despite reposition of the misplaced catheter detected by Dyna CT, and the main reason for repeated failure was difficult cannulation of the right adrenal vein.

The catheterisation of the right adrenal vein is the critical step in the entire AVS procedure, particularly for less-experienced operators. Dyna CT can provide three-dimensional anatomic information and aid in recognising the right adrenal venous orifice of the IVC. As the operator accumulates more experience, the confidence to locate the catheter on venography also increases, based on the past understanding via Dyna CT imaging. Although Dyna CT did increase the radiation exposure to the patient, the calculated additional dose (30 mGy) was only around 5% of the original AVS, which was 673.9  ±  613.8 mGy in this study^24^.

The present study has certain limitations. First, it was a retrospective study with a limited sample size. Second, all the procedures were performed by a single radiologist, which may not represent the general conditions in AVS procedures with Dyna CT. Third, the fluctuation of the patients’ hormone levels might influence selectivity results when the AVS is conducted without ACTH stimulation. Nevertheless, non-stimulated AVS is a well-established procedure for hyperaldosteronism lateralisation, and we implemented several measures for minimising patient stress during AVS in the present study. We believe that hormonal fluctuations were minimal and would introduce little bias to the study results.

In conclusion, our study showed that Dyna CT is useful to locate the catheter position in the right adrenal vein, and that the application of Dyna CT further increases the success rate of non-stimulated AVS.

## Additional Information

**How to cite this article**: Chang, C.-C. *et al*. Non-stimulated adrenal venous sampling using Dyna computed tomography in patients with primary aldosteronism. *Sci. Rep.* **6**, 37143; doi: 10.1038/srep37143 (2016).

**Publisher's note:** Springer Nature remains neutral with regard to jurisdictional claims in published maps and institutional affiliations.

**Publisher’s note:** Springer Nature remains neutral with regard to jurisdictional claims in published maps and institutional affiliations.

## Figures and Tables

**Table 1:** Patient characteristics between the successful adrenal venous sampling (AVS) group and the failed AVS group.

	Successful AVS (n = 87)	Failed AVS (n = 13)	*p* value
Age, years	52.2 ± 10.2	53.8 ± 10.2	0.609
Gender			0.772
Male, No. (%)	45 (51.7)	6 (46.2)	
Female, No. (%)	42 (48.3)	7 (53.8)	
Serum K^+^, mEq/L	4.1 ± 4.4	3.6 ± 0.7	0.715
Aldosterone, ng/dL	67.0 ± 112.7	55.1 ± 31.2	0.716
Renin activity, ng/mL/h	0.78 ± 1.26	0.68 ± 0.82	0.787
Body mass index, kg/m^2^	27.7 ± 4.3	25.7 ± 4.1	0.138
Adrenal nodule			0.692
None, No. (%)	23 (26.4)	3 (23.1)	
Right, No. (%)	20 (23.0)	2 (15.4)	
Left, No. (%)	29 (33.3)	4 (30.8)	
Bilateral, No. (%)	15 (17.2)	4 (30.8)	
Repositioning needed, No. (%)	12 (13.8)	4 (30.8)	0.215
Procedural time, min	49.1 ± 21.3	51.9 ± 21.9	0.66

**Table 2:** Comparison between the present study and the previous studies with similar purposes.

Authors	Auxillary Tools	No. of patients	Stimulation	Success rate of the right AVS, % (before/ after)	Determination of AVS success
Our study	Dyna CT	100	No	88/100	Selectivity index ≥2
Georgiades *et al*.^25^	C-arm CT	9	250 μg cosyntropin IV	78/100	appropriate increase of cortisol levels after cosyntropin stimulation (the precise level is N/A)
Plank *et al*.^14^	Dyna CT	19	No	Dyna CT detect 4 (21%) cases of misplacement	N/A
Park *et al*.^26^	C-arm CT	42	tetracosactide IV	86/95	Selectivity index ≥ 3
Higashide *et al*.^27^	Angio-CT	29	Yes, but the dose is not mentioned	76/97	Serum cortisol level >200 μg/dl in adrenal vein
Onozawa *et al*.^11^	Angio-CT	148	0.25 mg cosyntropin IV	87/96	Serum cortisol level >200 μg/dl in adrenal vein or selectivity index > = 5

## References

[b1] Rossi E. . High prevalence of primary aldosteronism using postcaptopril plasma aldosterone to renin ratio as a screening test among Italian hypertensives. Am. J. Hypertens. 15, 896–902 (2002). 12372677 10.1016/s0895-7061(02)02969-2

[b2] Rossi G. P. . A prospective study of the prevalence of primary aldosteronism in 1,125 hypertensive patients. J. Am. Coll. Cardiol. 48, 2293–2300 (2006). 17161262 10.1016/j.jacc.2006.07.059

[b3] Douma S. . Prevalence of primary hyperaldosteronism in resistant hypertension: a retrospective observational study. Lancet 371, 1921–1926 (2008). 18539224 10.1016/S0140-6736(08)60834-X

[b4] Kuo C. C. . Verification and evaluation of aldosteronism demographics in the Taiwan Primary Aldosteronism Investigation Group (TAIPAI Group). J. Renin Angiotensin Aldosterone Syst. 12, 348–357 (2011). 21393359 10.1177/1470320310391329

[b5] Lumachi F. . Long-term results of adrenalectomy in patients with aldosterone-producing adenomas: multivariate analysis of factors affecting unresolved hypertension and review of the literature. Am. Surg. 71, 864–869 (2005). 16468537

[b6] Group T. S. . Association of kidney function with residual hypertension after treatment of aldosterone-producing adenoma. Am. J. Kidney Dis. 54, 665–673 (2009). 19628318 10.1053/j.ajkd.2009.06.014

[b7] Rossi G. P. Diagnosis and treatment of primary aldosteronism. Rev. Endocr. Metab. Disord. 12, 27–36 (2011). 21369868 10.1007/s11154-011-9162-8

[b8] Magill S. B. . Comparison of adrenal vein sampling and computed tomography in the differentiation of primary aldosteronism. J. Clin. Endocrinol. Metab. 86, 1066–1071 (2001). 11238487 10.1210/jcem.86.3.7282

[b9] Young W. F. . Role for adrenal venous sampling in primary aldosteronism. Surgery. 136, 1227–1235 (2004). 15657580 10.1016/j.surg.2004.06.051

[b10] Funder J. W. . Case detection, diagnosis, and treatment of patients with primary aldosteronism: an endocrine society clinical practice guideline. J. Clin. Endocrinol. Metab. 93, 3266–3281 (2008). 18552288 10.1210/jc.2008-0104

[b11] Onozawa S. . Evaluation of right adrenal vein cannulation by computed tomography angiography in 140 consecutive patients undergoing adrenal venous sampling. Eur. J. Endocrinol. 170, 601–608 (2014). 24459237 10.1530/EJE-13-0741

[b12] Young W. F. Jr. & Klee G. G. Primary aldosteronism. Diagnostic evaluation. Endocrinol. Metab. Clin. North Am. 17, 367–395 (1988). 3042391

[b13] Auchus R. J. . Rapid cortisol assays improve the success rate of adrenal vein sampling for primary aldosteronism. Ann. Surg. 249, 318–321 (2009). 19212188 10.1097/SLA.0b013e3181961d77

[b14] Plank C. . Adrenal venous sampling using Dyna-CT–a practical guide. Eur. J. Radiol. 81, 2304–2307 (2012). 21620601 10.1016/j.ejrad.2011.05.011

[b15] Wu K. D. . Preoperative diagnosis and localization of aldosterone-producing adenoma by adrenal venous sampling after administration of metoclopramide. J. Formos Med. Assoc. 100, 598–603 (2001). 11695274

[b16] Wu V. C. . Kidney impairment in primary aldosteronism. Clin. Chim. Acta 412, 1319–1325 (2011). 21345337 10.1016/j.cca.2011.02.018

[b17] Chao C. T. . Diagnosis and management of primary aldosteronism: an updated review. Ann. Med. 45, 375–383 (2013). 23701121 10.3109/07853890.2013.785234

[b18] Kem D. C. . Circadian rhythm of plasma aldosterone concentration in patients with primary aldosteronism. J. Clin. Invest. 52, 2272–2277 (1973). 4353776 10.1172/JCI107414 PMC333030

[b19] Rossi G. P. . An expert consensus statement on use of adrenal vein sampling for the subtyping of primary aldosteronism. Hypertension 63, 151–160 (2014). 24218436 10.1161/HYPERTENSIONAHA.113.02097

[b20] Mulatero P. . Increased diagnosis of primary aldosteronism, including surgically correctable forms, in centers from five continents. J. Clin. Endocrinol. Metab. 89, 1045–1050 (2004). 15001583 10.1210/jc.2003-031337

[b21] Rossi G. P. . The Adrenal Vein Sampling International Study (AVIS) for identifying the major subtypes of primary aldosteronism. J. Clin. Endocrinol. Metab. 97, 1606–1614 (2012). 22399502 10.1210/jc.2011-2830

[b22] Seccia T. M. . Adrenocorticotropic hormone stimulation during adrenal vein sampling for identifying surgically curable subtypes of primary aldosteronism: comparison of 3 different protocols. Hypertension 53, 761–766 (2009). 19349554 10.1161/HYPERTENSIONAHA.108.128553

[b23] Mailhot J. P. . Adrenal Vein Sampling in Primary Aldosteronism: Sensitivity and Specificity of Basal Adrenal Vein to Peripheral Vein Cortisol and Aldosterone Ratios to Confirm Catheterization of the Adrenal Vein. Radiology 142413 (2015). 10.1148/radiol.2015142413 26020437

[b24] Georgiades C. . [Use of C-arm CT for improving the hit rate for selective blood sampling from adrenal veins]. Radiologe 49, 848–851 (2009). 19697002 10.1007/s00117-009-1865-4

[b25] Georgiades C. S. . Adjunctive use of C-arm CT may eliminate technical failure in adrenal vein sampling. J. Vasc. Interv. Radiol. 18, 1102–1105 (2007). 17804771 10.1016/j.jvir.2007.06.018

[b26] Park S. I. . Right adrenal venography findings correlated with C-arm CT for selection during C-arm CT-assisted adrenal vein sampling in primary aldosteronism. Cardiovasc. Intervent. Radiol. 37, 1469–1475 (2014). 24352864 10.1007/s00270-013-0820-y

[b27] Higashide T. . Detection of adrenal veins on selective retrograde CT adrenal venography in comparison with digital subtraction angiography in subjects with established diagnosis of one-sided adrenal aldosterone-producing tumor confirmed by adrenal vein sampling, histopathology and clinical course. Int J Cardiol. 168, 3254–3258 (2013). 23647597 10.1016/j.ijcard.2013.04.140

